# Unmet need for family planning and associated factors among currently married reproductive age women in Tiro Afeta District, South West Ethiopia, 2017: cross-sectional study

**DOI:** 10.1186/s12905-019-0872-5

**Published:** 2019-12-30

**Authors:** Tesfaye Solomon, Mamo Nigatu, Tsegaye Tewelde Gebrehiwot, Biniam Getachew

**Affiliations:** 1 West Shewa Zonal Health Department, Public Health Emergency Management Officer, P.O.Box 32, Ambo, Oromia Regional State Ethiopia; 2 Department of Epidemiology, Jimma University, Jimma, Ethiopia; 3 Independent Public Health Consultant, Addis Ababa, Ethiopia

**Keywords:** Unmet need, Family planning, Ethiopia

## Abstract

**Background:**

Unmet need for family planning in Oromia region was very high (28.9%) compared to other regions in Ethiopia. To address problems associated with unmet need for family planning locally available evidences are essential, however, there were no clear evidences on unmet need for family planning in Tiro Afeta district. This study aims to assess the magnitude and associated factors of unmet need for family planning among currently married women in Tiro Afeta district, South West Ethiopia, 2017.

**Methods:**

Community based cross sectional study was conducted in April, 2017. A total of 348 currently married women of reproductive age were enrolled from eight villages selected by simple random sampling and using proportional to size allocation. Data were entered using EpiData 3.1 and analyzed by SPSS version 22. Adjusted odds ratios at 95% confidence interval with *p*-value of < 0.05 were considered as significant variables.

**Results:**

Unmet need for family planning among currently married women in Tiro Afeta was 26.1%. Factors significantly associated with unmet need for family planning were: never use of family planning before survey (AOR: 5.09, 95% CI: 2.73–9.50); multiparity (AOR: 3.02, 95% CI: 1.56–5.85); perceived husband’s attitude as disapproval (AOR: 2.75, 95% CI: 1.43–5.26); lack of counseling from health workers (AOR: 2.07, 95% CI: 1.11–3.85); and unavailability of Radio and/or Television in the house (AOR: 2.05, 95% CI: 1.15–3.66).

**Conclusion:**

Unmet need for family planning in Tiro Afeta was higher than national average but lower than Oromia region. Never use of family planning, women’s parity, husband’s attitude towards contraceptives, women counseling and unavailability of Radio and/or Television in the respondent’s home were significantly associated factors with unmet need for family planning. Therefore, the service providers and the district health office should strengthen counseling and partner involvement to reduce unmet need for family planning.

## Background

Family planning (FP) is a means for improving health, reducing poverty, and empowering women [1–4]. It could prevent as many as one in every three maternal deaths by allowing women to delay motherhood, space births, avoid unintended pregnancies and abortions, and stop childbearing when they have reached their desired family size [5]. The ability of FP to reduce maternal deaths can be more realized if the poorest individuals and those with unmet need are reached on a wide scale [1, 6, 7].

The proportion of married women who are fecund and do not wish to become pregnant soon are assumed to have a “demand” for contraception [1, 5]. Married women of reproductive age (aged 15–49 years) have unmet need if they are fecund, do not want a child in the next 2 years or at all, and are not using any method of contraception, either modern or traditional. Pregnant women and women experiencing postpartum amenorrhea and who gave birth within 2 years prior to the survey are classified as having unmet need if they indicated that their current or recent pregnancy was unintended [8]. Women with unmet need fall into two groups: spacers and limiters [9].

Unmet need for FP shows the gap between women’s reproductive intentions and their contraceptive behavior. In principle, this indicator may range from 0 to 100. Unmet need levels of 25% or more are considered very high, and values of 5% or less are regarded as very low [10]. High levels of unmet need may indicate that women are not empowered to use contraception because they lack access to health care or are unable to negotiate FP with their partner or related to other factors such as cultural and/or religious factors [11].

Established by United Nations, the Sustainable Development Goal 3 targets to reduce the global maternal mortality ratio to less than 70 per 100,000 live births by 2030 [12]. Addressing the unmet need for FP would dramatically reduce maternal deaths due to preventable causes. Ethiopia is currently working towards reducing unmet need for FP from 22% in 2016 to 10% by the end of 2020 year, FP2020 goals [13].

Worldwide in 2015, 12% of married or in-union women are estimated to have had an unmet need for FP [1]. In six African countries, including Ethiopia about one-third of married women aged 15 to 49 years have an unmet need for a FP method [14]. About one in every three births is unintended in Ethiopia as consequence of unmet need [9]. These high levels of unintended pregnancies can pose serious health risks to mothers and their infants [15]. Reducing the unmet need would significantly reduce unintended pregnancies, abortions, and maternal and child deaths [16]. The unmet need for FP in Ethiopia is gradually declining from 36% in 2000 to 22% in 2016 [17]. In the Oromia region of Ethiopia, unmet need for FP was 31% in 2011 [18] and decreased to 28.9% in 2016, within a period of 5 years [17]. According to Tiro Afeta district health office report of 2016, the FP service coverage was 80% [19].

Studies conducted in Ethiopia on unmet need for FP so far were focused on larger towns in the country [20–23], hence this study provides evidence from a rural part of the country. To address problems associated with unmet need for FP, locally available evidences are essential for program managers and health administrators working at different levels. However, clear evidences on met and unmet need for FP are not available in the Tiro Afeta district. Therefore, the aim of this study is to determine magnitude of unmet need for FP and factors associated with it among currently married women in Tiro Afeta district, which will help program managers to tackle the problems in the study area; moreover, it provided evidence for other rural district in similar situation especially for developing countries.

## Methods

### Study setting

The study was conducted in the Tiro Afeta district of Jimma zone, Oromia Regional State which is located at a distance of 290 km from Addis Ababa to the South west of Ethiopia. The district has a total population of 156,425 of which 50% are female, and 34,883 are women of childbearing age group. The district has two urban and 23 rural kebeles (the smallest administrative unit, village). In the district, there are five health centers and 25 health posts providing family planning service to the community [19]. It has been a long time since intervention of family planning was implemented in the district but there was a paucity of evidence on met and unmet need of the service. Hence, the study area was selected to fill this gap.

### Study design and period

Community based cross sectional study design was conducted from sampled currently married reproductive age (15–49 years) women residing in the Tiro Afeta district at least for 6 months before the commencement of data collection. Women who were unable to respond, severely ill or not presented at home with two visits were excluded. Data were collected from April 1st -15th, 2017.

### Study variables

Dependent variable: Unmet need for family planning

The independent variables are as follows:

- Socio-Demographic factors: Age, religion, ethnicity, residence, occupation, respondent’s and husband’s education.
- Socio-economic variables: Household income, availability of radio and/or TV in the respondent’s home.
- Reproductive factors: Age at first marriage, number of pregnancy, number of living children, ever use of the family planning.
- Knowledge related factors: knowledge about FP, women’s approval of FP, perceived husband’s approval of contraception, discussion with husband about FP, visited HF in past 12 months and Counseled by HW/HEW on FP.

### Sample size calculation

The sample size was calculated by Epi-info version 7 for the unmet need for family planning using single population proportion formula, with the following assumptions: 95% CI, 5% margin of error and 28.9% unmet need for FP in Oromia region of Ethiopia [17]. Considering 10% non-response rate, the final sample was 348. The prevalence of unmet need gives the largest sample size than factors associated with unmet need for family planning and so used to determine the final sample size.

### Sampling techniques

Due to limitation of resource, eight villages (32%) out of 25 villages in the district were selected by random sampling. The total sample size was allocated proportionally to the total number of currently married reproductive age women in the sampled kebeles (the smallest administrative unit in the district). List of currently married reproductive age women were collected from family folder in the nearby health post and was used as a sampling frame for rural kebeles. In one urban kebele, a census was conducted to generate lists of currently married reproductive age women prior to data collection which used as a sampling frame. The simple random sampling technique was used to select currently married reproductive age women.

### Data collection instrument and techniques

A survey questionnaire adapted from the Demographic Health Survey 2012 [8] was used to collect data about the magnitude of and factor associated with unmet need for family planning. The structured questionnaire includes four sections. Section A include 14 items on socio-demographic characteristics; section B has 10 items on reproductive history; while section C has 19 items to filter for unmet need definition; section D has 10 items to measure knowledge and individual related factors on family planning; and section E include 6 items for assessing women’s/perceived husband’s approval of family planning. The questionnaire was originally prepared in English and translated into local language, Afan Oromo. To check for consistency, the questionnaires were further translated from Afan Oromo to English by another person.

The questionnaires were administered by the interviewer through face-to-face interview. Eight trained female nurses who can speak the local language (1 Nurse per Kebele) were recruited from other districts and supervised by three Health Officer and one BSc Nurse (1 Supervisor per 2 data collectors) from the catchment health center.

### Data quality assurance

Data collectors were provided with one-day training about the objective, process of data collection and field ethics. Closer supervision was conducted during data collection. Each questionnaire was checked daily by the supervisors and the principal investigator. Pretest was conducted in Afeta kebele (non sampled kebele of the district) taking 5% of the total sample. Consequently, clarifications and corrections were made on vague points, jargon questions and other problems encountered about the questionnaire.

### Data analysis

Each questionnaire was coded and checked manually for completeness and consistency. Then, data were entered into Epidata 3.1 and exported to SPSS version 22 where recoding, categorizing, computing, counting and other statistical analysis were done. Univariate analysis like measures of central tendency and measures of dispersion for continuous variables was computed. Frequency distribution was done for categorical data. Bivariate analysis was done to select candidate variables with *P* < 0.25. Then entered into multivariable analysis to identify independent predictor variables and control for confounders. Multicolinearity was checked by using Variance Inflation Factor (VIF) and no problems were identified (VIF < 10). Model adequacy was checked by Hosmer & Lemeshow goodness of test (*p*-value > 0.05). In multivariable logistic regression, Adjusted Odds Ratio (AOR) with its 95% Confidence Interval (CI) was computed for variables maintained in the final model and statistical significance was declared by the confidence interval.

### Operational definition

**Knowledge:** Each knowledge question answered correctly was scored one mark while question answered incorrectly was scored zero mark. The total score ranging from 0 to 10 obtained by each respondent was added up and the mean was computed to categorize knowledge.

**Good knowledge:** when a woman correctly answered above the mean out of ten knowledge questions administered (i.e. greater than or equal to eight).

**Poor knowledge:** when a woman correctly answered below the mean out of ten knowledge questions administered (i.e. less than or equal to seven).

## Results

### Socio-demographic characteristics

A total of 348 (100%) study subjects participated in the survey. The mean age of the respondents was 29.6 ± 6.7 years and the age ranges from 15 to 49 years. The highest proportion of the respondents 168 (48.3%) were in the age group 25–34 years and 308 (88.5%) were residing in rural areas. Two third of respondents and their husbands were illiterate, 64.7 and 64.9% respectively. The majority of respondents (93.1%) were Oromo in ethnicity and Muslim, 294 (84.5%), in religion. The monthly income of the majority of the respondents (84.5%) was < 1500 (see Table 1).

**Table 1:** Socio demographic characteristics of currently married women in Tiro Afeta district, South West Ethiopia, May, 2017

Characteristic	Categories	Number (%)	Percentage of unmet need for FP
Residence	Rural	308 (88.5)	85 (27.6%)
	Urban	40 (11.5)	6 (15.0%)
Age	15–24	83 (23.9)	21 (25.3%)
	25–34	168 (48.3)	37 (22.0%)
	35–49	97 (27.9)	33 (34.0%)
Religion	Muslim	294 (84.5)	85 (28.9%)
	Orthodox	39 (11.2)	6 (15.4%)
	Protestant	15 (4.3)	0 (0.0%)
Ethnicity	Oromo	324 (93.1)	87 (26.9%)
	Amhara	11 (3.2)	0 (0.0%)
	Other*	13 (3.7)	4 (30.8%)
Women education	Illiterate	225 (64.7)	69 (30.7%)
	Primary	73 (21.0)	18 (24.7%)
	Secondary & above	50 (14.4)	4 (8.0%)
Husband education	Illiterate	226 (64.9)	71 (31.4%)
	Primary	61 (17.5)	15 (24.6%)
	Secondary & above	61 (17.5)	5 (8.2%)
Occupation	Housewife	272 (78.2)	84 (30.9%)
	Merchant	30 (8.6)	4 (13.3%)
	Gov’t employer	28 (8.0)	1 (3.6%)
	Other**	18 (5.2)	2 (11.1%)
Household Monthly income	< 1500	294 (84.5)	88 (29.9%)
	1500–3499	41 (11.8)	2 (4.9%)
	> 3500	13 (3.7)	1 (7.7%)
Availability of Radio and/or TV in the home	Yes	201 (57.8)	37 (18.4%)
	No	147 (42.2)	54 (36.7%)

Other* = Yem 9, Dawuro 2 and Tigre 2

Other** = student 6, jobless 4, daily laborer 4 and local drink seller 4

### Unmet need for family planning

**Fig. 1:**
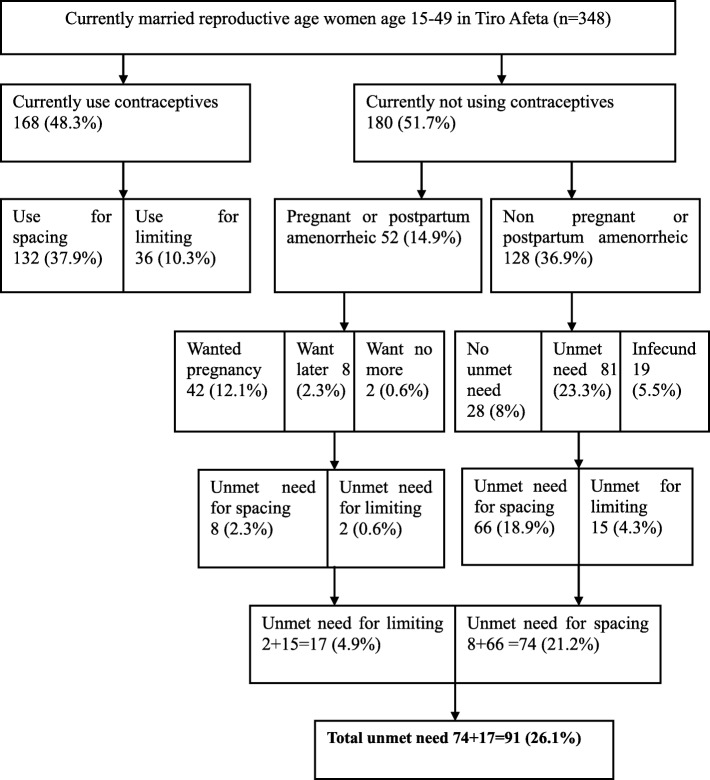
Unmet need of family planning among currently married women in Tiro Afeta district, South West Ethiopia, May, 2017 (Westoff Model, 2012)

Further analysis was carried out on the currently married women of 15–49 years to determine the level of unmet need for family planning by using the Westoff model [8]. The total unmet need of contraceptives in the Tiro Afeta district was found to be 91 (26.1%) of which 74 (22.2%) for spacing and 17 (4.9%) for limiting (see Fig. 1). The total demand for contraception was 74.4% (current use 48.3% + unmet need 26.1%). Of these, 64.1% had satisfied demand with modern contraceptives. The unmet need for modern methods was 26.7%.

### Reproductive history of the respondents

The mean age of women at marriage was 18.5 ± 2.4 SD years. Majority 316 (90.8%) of the respondents experienced at least one and above pregnancy. The average number of pregnancies per woman was 4.47 ± 2.63 SD. Thirty-six (10.3%) of the respondents had a history of spontaneous or induced abortion, of which three (8.3%) experienced more than one spontaneous abortion in their lifetime. The average number of children per woman was 4.04+ 2.43. Among reproductive factors, number of living children and ever use of family planning were nominated variables for the multivariable analysis (see Table 2).

**Table 2:** Reproductive history among currently married women in Tiro Afeta district, South West Ethiopia, May, 2017

Characteristic	Category	Frequency (%)	Unmet need for FP		COR (95% CI)	P-Value
			Yes	No		
Age at first marriage	< 18	114 (32.8)	31	83	1.08 (0.65–1.80)	0.757
	> 18	234 (67.2)	60	174	1	
Number of pregnancy	< 2	127 (36.5)	29	98	1	
	3–4	83 (23.9)	17	66	0.87 (0.44–1.71)	0.687
	> 5	138 (39.7)	45	93	1.64 (0.95–2.82)	0.078
Number of alive children	< 2	150 (43.1)	31	119	1	
	3–4	82 (23.6)	18	64	1.08(.56–2.08)	0.819
	> 5	116 (33.3)	42	74	2.18 (1.26–3.77)	0.005
History of abortion	Yes	36 (10.3)	12	24	1.48 (0.71–3.09)	0.303
	No	312 (89.7)	79	233	1	
Experience of stillbirth/child loss	Yes	67 (19.3)	18	49	1.05 (0.57–1.91)	0.882
	No	281 (80.7)	73	208	1	
Ever used of contraceptives before the survey	Yes	227 (65.2)	30	197	1	
	No	121 (34.8)	61	60	6.68 (3.95–11.27)	< 0.001

### Knowledge of the respondents

Regarding the knowledge status of the respondents, 229 (65.8%) had good knowledge about family planning. Among those who had poor knowledge, 54(45.3%) had an unmet need for family planning. Most of the respondents 342 (98.3%) heard at least one method of contraceptives from which 304 (88.9%) knew injectable contraceptive followed by 292 (85.4%) and 270 (78.9%) who knew oral contraceptive pills and implants, respectively. Those women who heard about any contraceptive also had heard that the sources of modern contraceptives were health facilities in the district. Of these, 310 (90.6%) and 285 (83.3%) of the respondents mentioned health posts and health centers, respectively, as the source of modern contraceptives for themselves and/or other women.

The majority 294 (96.8%) of the respondents heard information about family planning from health workers (health professionals and HEWs) followed by Radio and/or TV 225 (65.8%) and friends 108 (31.6%). Two hundred one (57.8%) of the respondents had media (radio and/or television) in their home.

In the past 6 months before the initiation of data collection, 203 (58.3%) women discussed about contraceptive methods with their partner at least once. Among these, 141 (40.5%) discussed once/twice and 49 (14.1%) respondents have discussed about contraceptive methods more often with their husband. About 44% of women who had an unmet need for family planning reported that their partners disapproved/opposed them to use family planning, while 234 (67.2%) reported that their husband’s approved family planning and 34 (9.8%) didn’t know their husband’s attitude towards family planning. More than two-third 243 (69.8%) of the respondents visited health facilities at least once in the past 12 months before data collection. Among these 163 (46.8%) of the respondents were counseled about methods of contraception by health professionals.

### Predictors of unmet need for family planning

Multivariable analysis was carried out to determine independent predictors of unmet need for family planning. Those candidate variables with *P*-value < 0.25 in bivariate logistic regression were included in multivariable logistic regression and considered as significant in the model if the P-value less than 0.05.

Previous use of family planning method was found to be an independent predictor of unmet need for family planning. Currently married women who never used family planning method previously were found five times more likely to had an unmet need for family planning compared to those women who ever used family planning method (AOR: 5.09, 95% CI: 2.73–9.50).

Parity was found to be an independent predictor of unmet need for family planning. Multiparous women were found three times more likely to have had an unmet need for family planning compared with those women who had two and less living children (AOR: 3.02, 95% CI: 1.56–5.85).

Women whose husbands disapprove family planning were found more than two times more likely to have had unmet need for family planning than those respondents whose husbands approved family planning (AOR: 2.75, 95% CI: 1.43–5.26).

Furthermore, women’s reports of whether they were counseled or not to use family planning methods were found to be an associated factor of unmet need for family planning. Women who were not counseled by health personnel’s were two times more likely to have had unmet need for family planning than those women who get counseled by health personnel in the past 12 months (AOR: 2.07, 95% CI: 1.11–3.85).

Availability of media (Radio and/or TV) in the house was a factor associated with unmet need for family planning. Those respondents who had no Radio and/or TV in their house were found two times more likely to have unmet need for family planning than those women who have had TV and/or Radio in their home (AOR: 2.05, 95% CI: 1.15–3.66) (see Table 3).

**Table 3:** Independent predictors of unmet need for family planning among currently married women in Tiro Afeta district, South West Ethiopia, May, 2017

Variables	Category	Number (%)	Unmet need for FP		Crude OR (95% CI)	Adjusted OR (95% CI)
			Yes	No		
Available Radio and/or TV	Yes	201 (57.8)	37	164	1	1
	No	147 (42.2)	54	93	2.57 (1.58–4.20)	2.05 (1.15–3.66)*
Number of alive children	< 2	150 (43.1)	31	119	1	1
	3–4	82 (23.6)	18	64	1.08 (0.56–2.80)	1.38 (0.64–2.95)
	> 5	116 (33.3)	42	74	2.18 (1.30–3.77)	3.02 (1.56–5.85)*
Perceived husband’s attitude towards FP	Approve	234 (67.2)	38	196	1	1
	Disapprove	80 (23.0)	40	40	5.16 (2.95–9.02)	2.75 (1.43–5.26)*
	Don’t know	34 (9.8)	13	21	3.19 (1.47–6.92)	0.90 (0.35–2.30)
Counseled about FP	Yes	163 (46.8)	23	140	1	1
	No	185 (53.2)	68	117	3.54 (2.08–6.03)	2.07 (1.11–3.85)*
Ever use of FP methods	Yes	227 (65.2)	30	197	1	1
	No	121 (34.8)	61	60	6.68 (3.95–11.27)	5.09 (2.73–9.50)*

Note: *P < 0.05 (Indicates statistically significant)

In the final logistic regression model education of women and her husband, number of pregnancies, couples approval and discussion of family planning, knowledge status and health facility visit were not associated with unmet need for family planning.

## Discussion

This study was conducted to determine the level of unmet need for family planning and identify associated factors among currently married women. This study revealed the magnitude of unmet need for family planning in the Tiro Afeta district was very high, 26.1%, which was above the national average (22%) [17]. The level of unmet need for family planning in the district was above other community based studies in Ethiopia, to mention some, Tigray (21.1%) [21] and Amhara (17.4%) [22] regions; but below the level of unmet need found in Butajira district (52%) [23] and Oromia regional levels (28.9%) [17]. This result is also far from the national target of reducing the level of unmet need for FP to 10% by 2020 [13]. This indicates that even though there was improvement in contraceptive prevalence among currently married women in the district; achieving the target and maximizing the benefits of FP requires dedication to provide FP to those women with the identified unmet need.

The contraceptive prevalence rate among currently married reproductive age women in the area was 48.3% which is above the national figure of EDHS, 2016 (22%) [17]. This might be due to the provision of family planning services at the community level through health extension workers and health professionals. However, the service might be not effective in reaching those married women who demonstrated unmet need for FP.

In this study, parity was found to be one of associated factors of unmet need for family planning. Women who had five or more alive children were three times more likely to have unmet need for family planning when compared to those who had two or less alive children or no child alive at all (AOR: 3.02, 95% CI: 1.56–5.85). This result agrees with the results of other studies [21, 24, 25] those showed women who had few children (two or less) were more likely use family planning compared to women with five or more children. These might be due to the more children the woman is having; the more likely she wants to space or limit the number of children she will have. This implies the more she was not using family planning and had an unmet need.

Women whose partner did not approve using family planning were found more than two times more likely to had an unmet need for family planning than those respondents whose husbands approve family planning (AOR: 2.75, 95% CI: 1.43–5.26). This result is similar to a study done in Tigray [21]) and it indicates that male involvement in family planning could be a factor in increasing the number of family planning users. Other studies done in Kenya and Ethiopia found that partner approval [26] and husband support [27] influences the use of FP services and contraceptives respectively. The finding of this study may indicate that women are not empowered to use contraception because they are unable to negotiate family planning with their partner.

Women’s previous use of family planning was also found to be an independent predictor of unmet need for family planning. The odd of having an unmet need for family planning among currently married women who never used FP methods before was five times more likely than the odds of unmet need for FP among married women who ever used family planning before (AOR: 5.09, 95% CI: 2.73–9.50). This is similar to studies done in Debre Markos [28] and Uganda [29]. This tells us ever users of family planning have had an awareness to accept contraceptives and they were less likely to have had an unmet need for family planning.

This study also revealed that women who were not counseled by health personnel’s to use family planning methods were found two times more likely to have had an unmet need for family planning than those women who were counseled (AOR: 2.07, 95% CI: 1.11–3.85). This finding is similar to other studies that showed women not counseled by health professionals were more likely to have unmet need for contraceptives [20, 22]. Another study showed that an integration of FP services for female clients with frequently used Maternal and Child Health and reproductive services would lower costs to clients and reduce missed opportunities for service delivery [30]. This implies that family planning services can be made more accessible and convenient to clients if they take into account other service needs of the potential clients.

Availability of Radio and/or TV in the respondents’ home was also an associated factor with unmet need for family planning. Women who had no media (TV and/or Radio) in their homes were two times more likely to have unmet need for family planning compared to those women who had Radio and/or TV (AOR: 2.05, 95% CI: 1.15–3.66). This finding is in line with a further analysis of EDHS [31], that found women who have media exposure are significantly less likely to have an unmet need for spacing and limiting. Another study [32] reported that unmet need for family planning is positively associated with exposure to family planning messages. The finding of this study indicates that women who had no media in their home need awareness creation activities to deal with unmet need for family planning among these women.

This study found no association with urban-rural residence. This result is not similar to other studies that showed the rural residence is positively associated with unmet need for family planning [23, 25, 32]. This might be due to equal accessibility of family planning options for both urban and rural at community levels in the district.

## Limitation

Social desirability bias and recall bias might be introduced. Because, those pregnant and post-partum amenorrheic women were asked about their current and recent pregnancy respectively. Therefore, they might not exactly remember or sometimes hide the wontedness or planned time of their pregnancy. To minimize these, vital events in women’s life were asked, and female data collectors were recruited.

This study was limited to married women only and therefore will not be generalized to unmarried women with unmet need for family planning in the general population.

## Conclusions

Unmet need for family planning in the Tiro Afeta district was high. Factors significantly associated with unmet need for family planning were women parity, previous use of family planning, women counseling, husband’s attitude towards FP and availability of Radio and/or TV in the respondents’ house. Therefore, family planning service providers should strength counseling on a wide variety of family planning choices and should give special attention to those women who never used FP, multiparous and those women who had no media in their home. The district health office should promote the involvement of men in family planning service through community meetings and panel discussions.

## Data Availability

The data supporting our findings are found at, kept in confidentiality and stored at the corresponding author both in hard and soft copies. If someone wants our data, we are voluntary to share it and the corresponding author should be contacted through the email address on the cover page.

## Abbreviations

AOR: Adjusted Odds Ratio
CI: Confidence interval
ETB: Ethiopia Birr
FP: Family Planning
HEW: Health Extension Workers
HF: Health Facility
HW: Health Workers
TV: Television
